# Patent landscape analysis—Contributing to the identification of technology trends and informing research and innovation funding policy

**DOI:** 10.1111/1751-7915.14201

**Published:** 2023-01-24

**Authors:** Tomas van Rijn, James Kenneth Timmis

**Affiliations:** ^1^ Netherlands Patent Office The Hague The Netherlands; ^2^ Athena Institute for Research on Innovation and Communication in Health and Life Sciences Vrije Universiteit Amsterdam Amsterdam The Netherlands

## Abstract

Patents and the systematic analysis thereof provide important decision information for a range of stakeholders pursuing diverse goals. However, in particular academia and small‐scale private sector innovators underappreciate the value of patent information for identifying research gaps, ensuring originality of their work and, in turn, maximisation of limited (public) funds. By the same token, pertinent public sector organisations, such as regulators, require overviews of potentially upcoming technologies to adequately adapt regulatory protocols. The latter, in particular if contemporary scientific evidence is not sufficient, can require a substantial amount of time and lead to a delay in the marketing of important innovations. Patent landscape analysis (PLA) is a very useful method to create overviews of technology fields and thereby indicate if, inter alia, a specific line of scientific enquiry and its application have already been pursued or potential regulatory gaps will exist in the near‐ to mid‐term future. The objective of this communication is to increase awareness of the utility of patent information and provide support in retrieval and analysis of pertinent information for those involved in biotech R&D. Based on Espacenet, a patent search engine, we provide basic guidance on search strategy development, piloting and execution, and data preparation and analysis. To highlight the value of PLA, we also summarise selected results of a PLA we performed recently for microbiome targeting interventions, also referred to as live biotherapeutic products.

## Graphical abstract

Patents and the systematic analysis thereof provide important decision information for a range of stakeholders pursuing diverse goals. Patent landscape analysis (PLA) is a very useful method to create overviews of technology fields and thereby indicate if, inter alia, a specific line of scientific enquiry and its application have already been pursued or potential regulatory gaps will exist in the near‐ to mid‐term future. Based on Espacenet, a patent search engine, we provide basic guidance on search strategy development, piloting and execution, and data preparation and analysis.

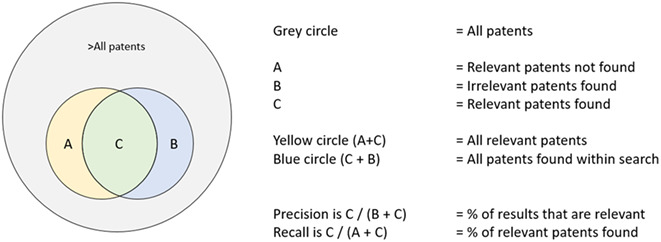

## INTRODUCTION

The start of new, or changes to existing, research and development projects, in most cases, entails the commitment of substantial resources, be this by public funders, private sector investors, etc. While academia has an obligation to maximise the utility of public funds for the benefit of society, private sector innovators, although not obliged to maximise benefits for society, are committed to their shareholders or investors to maximise potential financial rewards. In addition, establishing or adapting regulatory frameworks to include relevant scientific evidence and meeting pertinent market needs can be a lengthy procedure (Bubela et al., 2013). Failing to put in place regulatory protocols and guidance in a timely manner can lead to important mismatches between demand and supply, and runs the risk of postponing or preventing the introduction of important and innovative techniques, products and services, such as new health technologies and other biotechnology applications. While new R&D projects are frequently based on (good) ideas, diligent analysis of all sources of existing technology concepts/technologies is not always the case, which can lead to a ‘re‐invention of the wheel’ and the unnecessary waste of limited resources. Accessing existing academic and grey literature as well as clinical trial registers, is often a helpful starting point for identifying existing research and technologies, however, such documents do not contain information disclosed in patent documents that cover inventions that might not be described in other resources. Increasingly, patent landscape analysis is being recognised as a sound research method that can provide – on its own or in combination with other sources – various stakeholders with important decision information (Bonino et al., 2010).

**Table:** 

**Useful resources**
I – Guidelines for preparing patent landscape reports, World Intellectual Property Office. 2015. https://www.wipo.int/publications/en/details.jsp?id=3938
II – OECD Patent statistics manual. Organisation for Economic Co‐operation and Development. 2009. https://www.oecd.org/sti/inno/oecdpatentstatisticsmanual.htm
III – The Reporting Items for Patent Landscapes statement ‐ https://pubmed.ncbi.nlm.nih.gov/30412195/
IV – Espacenet – Pocket guide. European Patent Office., 2022. https://documents.epo.org/projects/babylon/eponet.nsf/0/8C12F50E07515DBEC12581B00050BFDA/$File/espacenet‐pocket‐guide_en.pdf
V – Patlib centres: https://www.epo.org/searching‐for‐patents/helpful‐resources/patlib.html

A patent landscape analysis (PLA) investigates patent applications in specific technology fields with the aim of identifying pertinent developments over time. A variety of patent analytical methods exist. Novelty or patentability searches, freedom to operate or validity searches, are common types of patent searches, which are usually more pertinent to answering questions regarding commercial activities based on own/third party patents. PLA, the discussion of which is the focus of this communication, is more closely related to acquiring market insights on a national or regional level, or across organisations.

Depending on specific PLA research objectives and the level of detail of pertinent analyses, PLAs can provide not only policy makers and regulators but also other important stakeholders, such as academia and commercial enterprises, with data on changes and trends in and across groups of technologies. By producing recent overviews of the technologies that public and private R&D organisations consider valuable, and therefore worth protecting against unauthorised replication and/or utilisation, PLAs can indicate hitherto unpursued lines of enquiry, and potential near‐ to mid‐term technology regulation needs and R&D blind‐spots. This, in turn, can support, for instance, identification of regulatory evidence gaps that can be addressed by prioritised/earmarked research funds and can be further explored by appropriate research organisations. In addition, including patent information early in the R&D process can improve focus of R&D efforts (and thereby prevent ‘reinventing the wheel’), and incentivise organisations to address hitherto unsolved technical challenges, or improve existing technologies. While commercial parties might focus on competitor or market analysis, public parties, such as policy makers, funding agencies and academia, will likely be more interested in economic performance and societal impact (Bonino et al., 2010; Bubela et al., 2013; Cordaillat‐Simmons et al., 2020; Timmis et al., 2021; Trajtenberg & Jaffe, 2002; Trippe, 2015).

The objective of this brief communication is to enhance awareness of the value of patent information and reduce challenges in retrieving pertinent information for individuals and organisations involved in biotech R&D, and bringing innovations to market.^1^ We (i) provide a hands‐on, entry‐level introduction including pointers and helpful suggestions on patent landscape analysis for determining patenting activity trends and (ii) showcase the utility of this method by providing a brief overview of selected results for microbiome targeting interventions—also referred to as live biotherapeutic products; published elsewhere: (Timmis et al., 2022).

## PATENT LANDSCAPE ANALYSIS: BACKGROUND KNOWLEDGE

Patent landscape analyses are per definition retrospective studies, because the overwhelming majority of patent documents are published 18 months after initial application—PLAs are based on published patent documents. However, as patents are usually filed long before related products enter the market, they are often also an early indicator of upcoming technologies. The initial patent application is referred to as the *priority filing* and the corresponding application date as the *priority date*, respectively. Once the priority filing has been submitted, further patent applications for the identical invention—provided, the substance of these applications is deemed technically identical vis‐à‐vis the priority filing—can be submitted in other jurisdictions, that is to other patent offices, over the course of one year (*priority year*). Together, the priority filing and subsequent filings for the same invention submitted within the priority year are called a *simple patent family* (Martínez, 2011). All documents within a particular patent family—such as the initial application, grants and others—are referred to as *publications* (Dechezleprêtre et al., 2017).

Good practice guidelines exist for executing and/or appraising, and reporting patent landscape analyses, as is the case for most types of scientific enquiry. The importance of rigorously consulting these sources for a priori determination of adequate study design, data collection and analysis (see e.g. Bubela et al., 2013) and proper reporting (see e.g. Smith et al., 2018) cannot be overstated, in particular for scholars (and other analysts) executing PLA for the first time. A great overarching resource for the identification of appropriate reporting guidelines—which, in turn, also supports the identification of literature describing good practice study design, execution and appraisal—can be found at https://www.equator‐network.org (Centre for Statistics in Medicine, 2022). *NB*: unintuitively, the search function is located in the bottom left corner of the website.

In essence, PLAs are meta‐analysis of patent data associated with specific variables such as *priority date* (date of initial patent application submission), *applicants or inventors* and their pertinent country of origin, patent technology classifications like *IPC* (*International Patent Classification*) and *CPC* (*Cooperative Patent Classification*) and so on, see below and in Glossary. PLAs are, therefore, quite similar to meta‐analyses conducted frequently within the context of systematic literature reviews. Depending on the type of information that is sought, *single field* patent analysis—the most basic form of analysis—can be used to curate overview lists and/or rankings of a group/body of patents based on individual variables, such as the above‐mentioned examples. *Matrix analyses* goes one step further by exploring associations within a body of records across said variables. Thereby, potential relationships between, for example

- technology classifications (classes)
- organisations
- locations of R&D
- markets/regions potentially considered to be of particular value for certain organisations/technologies
- and other patent/technology characteristics

can be identified and resulting record clusters and trends investigated (Clarke & Jürgens, 2019; Jürgens & Clarke, 2019; Ramezanpour et al., 2015; Smith et al., 2018; Van de Burgwal et al., 2016; Van Dongen et al., 2017).

A cornerstone of PLA are patent technology classifications such as the above mentioned IPC and CPC codes. These classifications aid in finding and clustering patents based on specific technologies described therein. CPC codes are a more recent extension of IPC codes and thus represent a more nuanced classification system. Classifications are tiered from broadest to narrowest concept coverage: section, class, subclass, main group, subgroup. Table 1 shows a classification example for drugs specifically addressing Parkinson's disease.

**TABLE 1:** Classification tree of A61P25/16.

Classification tier	Symbol	Description
Section	**A**	Human necessities
Class	A**61**	Medical or veterinary science; hygiene
Subclass	A61**P**	Specific therapeutic activity of chemical compounds or medicinal preparations
Main group	A61P**25/00**	Drugs for disorders of the nervous system
Sub‐group	A61P25/**14**	• for treating abnormal movements, e.g. chorea, dyskinesia
Subgroup^a^	A61P25/**16**	• • Anti‐Parkinson drugs

^a^ The tier depth, depicted by the number of dots (•), that is number of subgroups, varies depending on the classification.

**FIGURE 1:**
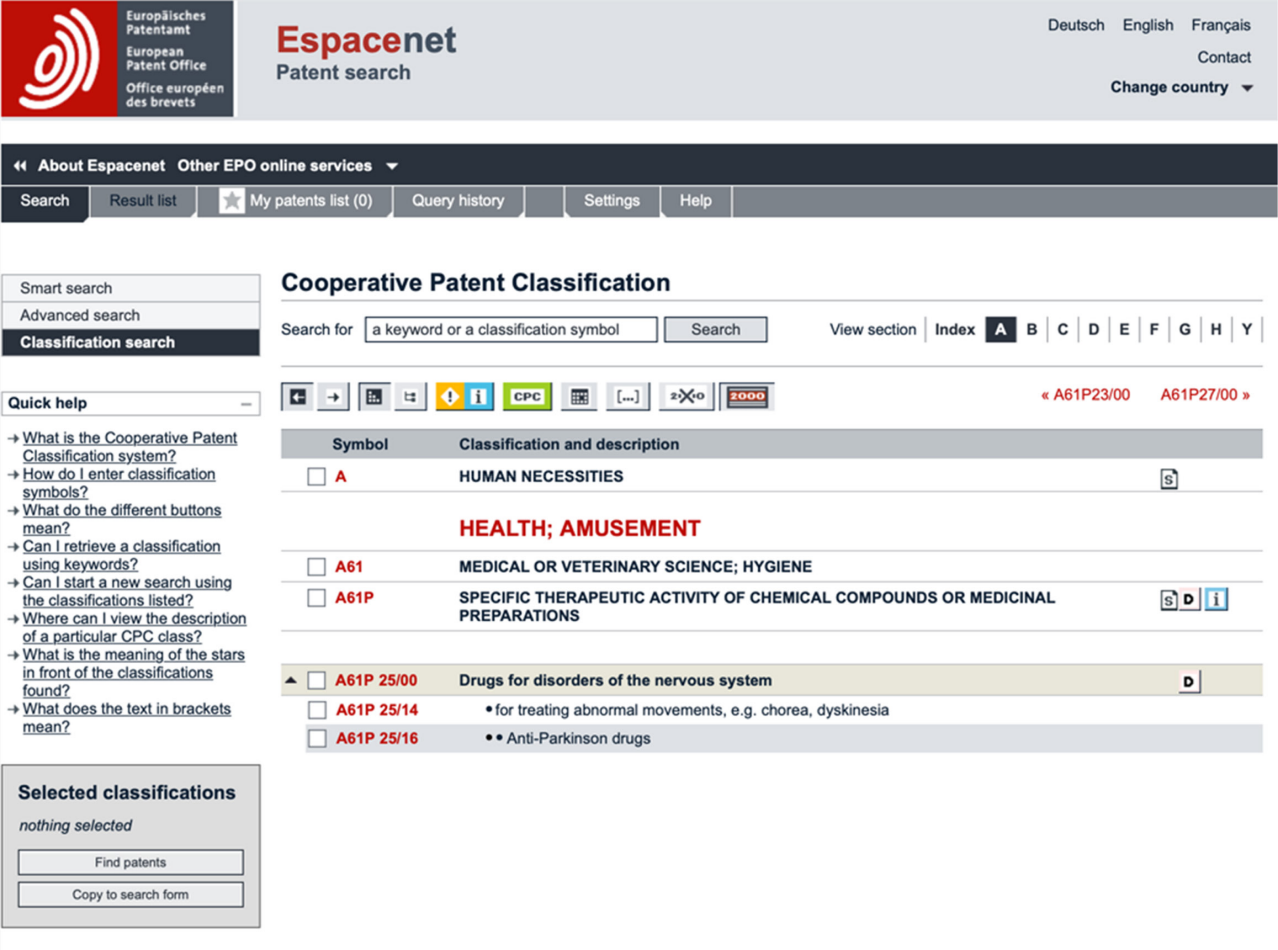
Classification tree of A61P25/16 on the Espacenet patent classification browser (screen capture).

For visualisation purposes (corresponds with Table 1), Figure 1 shows what the classification tree of A61P25/16 looks like on the Espacenet patent classification browser (https://worldwide.espacenet.com/classification). Note: A61P25**/16** is a further (but the only additional) specification of A61P25/14 (*14*: abnormal movements, e.g. chorea, dyskinesia). This means that patents associated with the superordinate classification level (A61P25**/14**) cover any medicine addressing abnormal movement disorders, and not only those based specifically on Parkinson's disease.

Classifications and their levels often play an important role in PLAs, as they allow translation of keyword‐based searches into more objective, classification‐based searches—bypassing language and terminology issues.

## FINDING PATENT DATA

There exist various online databases to query patent information.^2^ Many are hosted by commercial organisations, feature extensive search and filtering capabilities and—often as a basis for business analytics—secondary analyses including, for example detailed forward citation or textual claims analysis of patents (and/or patent families) and, thereby, not seldom, cater to the needs of the private sector. While these types of granular analyses can be relevant to research focussing on, for example the evolution and/or value of a specific invention or patent filing organisation, PLAs are, as alluded to above, based on patent meta‐data and, as access fees to commercial databases can be high, such databases might not be suitable for academic PLA.

A more affordable alternative is PATSTAT, the database hosted by the European Patent Office (EPO). While PATSTAT's options for visualisation of patent data are limited (compared to many other commercial databases), it is designed specifically for the purpose of statistical analysis. However, knowledge of SQL is required. Once familiarity with the system has been established, the data output can be easily exported to Excel or a database managements system (DBMS). The Lens, a more recently launched database, is also worth exploring as it provides individual records but also interesting integrated analysis of patents with other sources of information, such as academic literature. What might be of particular interest to the readership of Microbial Biotechnology, The Lens PatSeq also provides the capability to “search and analyze biological sequences disclosed in patent literature” (The Lens, 2022). As the above‐mentioned databases can be costly for individuals (and research groups), it is worth enquiring with university libraries or knowledge/technology transfer offices if licence agreements for patent databases exist. The Lens might be an attractive option among the subscription‐based databases.

Free and easily accessible patent information databases also exist, such as Espacenet, which is also hosted and maintained by the European Patent Office (EPO). Espacenet is a comprehensive patent search engine affording access to (and thereby the meta‐data of) 140 million patents that have been submitted to more than 90 patent offices worldwide (European Patent Office, 2022). Individual records and their claims can be reviewed, basic statistics of the retrieved body of records displayed for quick reference—post search (see below)—and pertinent meta‐data downloaded.

Although both The Lens and Espacenet are user‐friendly and provide helpful tools, they are not designed per se for statistical analyses. For instance, they have limited export volumes: 10,000 and 500 records per export, respectively (for Espacenet, we explain how to deal with this limitation below). Nevertheless, Espacenet's patent coverage, user‐friendliness, free access to, and good visualisation of, patent data, render it a particularly helpful tool for explaining (and performing) basic PLAs, which is also why, in the following sections, our accounts are based primarily on Espacenet: https://worldwide.espacenet.com/

## APPROACH TO A PATENT SEARCH^3^

**FIGURE 2:**
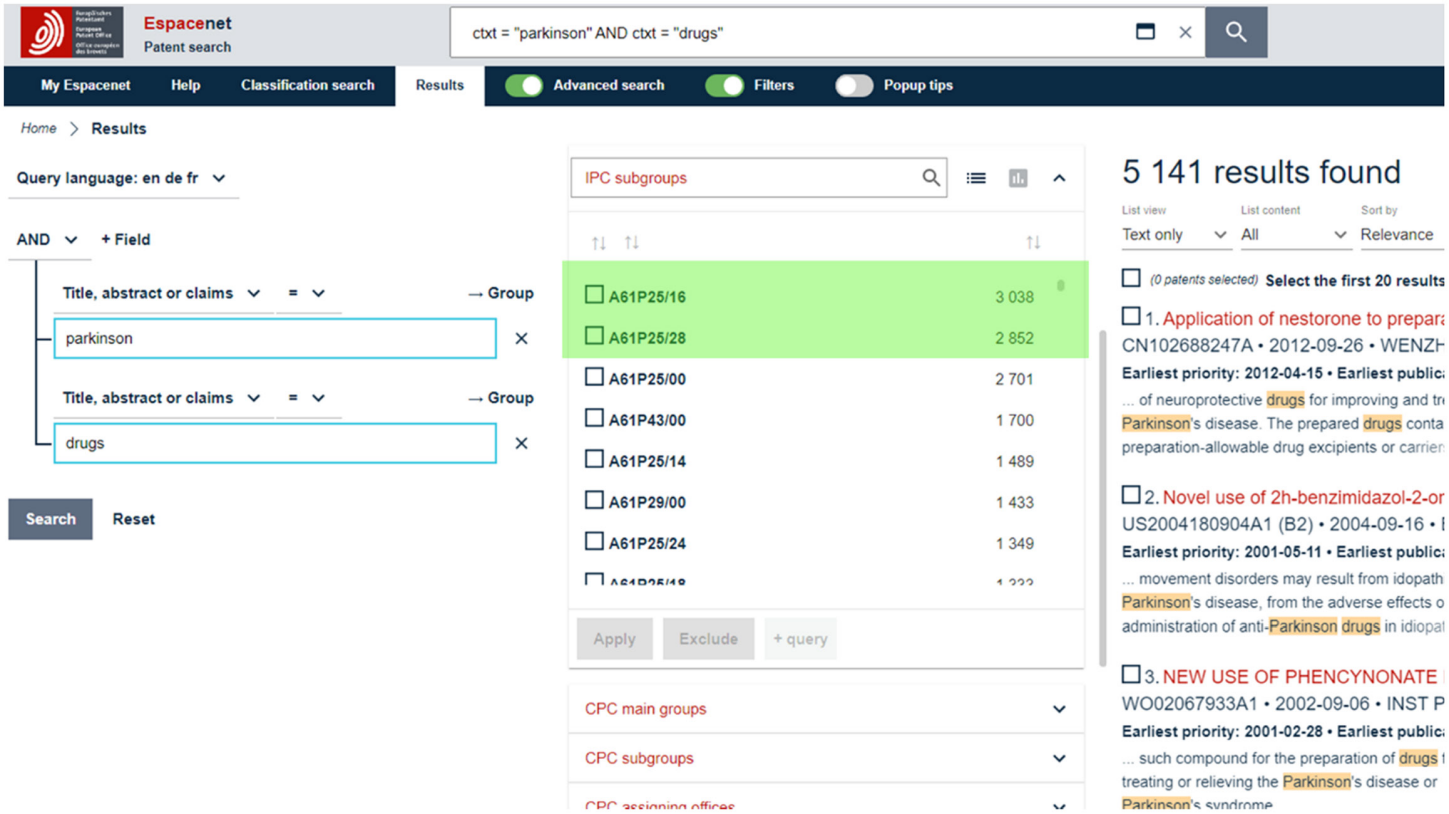
A *T* *itle, abstract or claims* search for *Parkinson AND drug* on Espacenet (screen capture).

**FIGURE 3:**
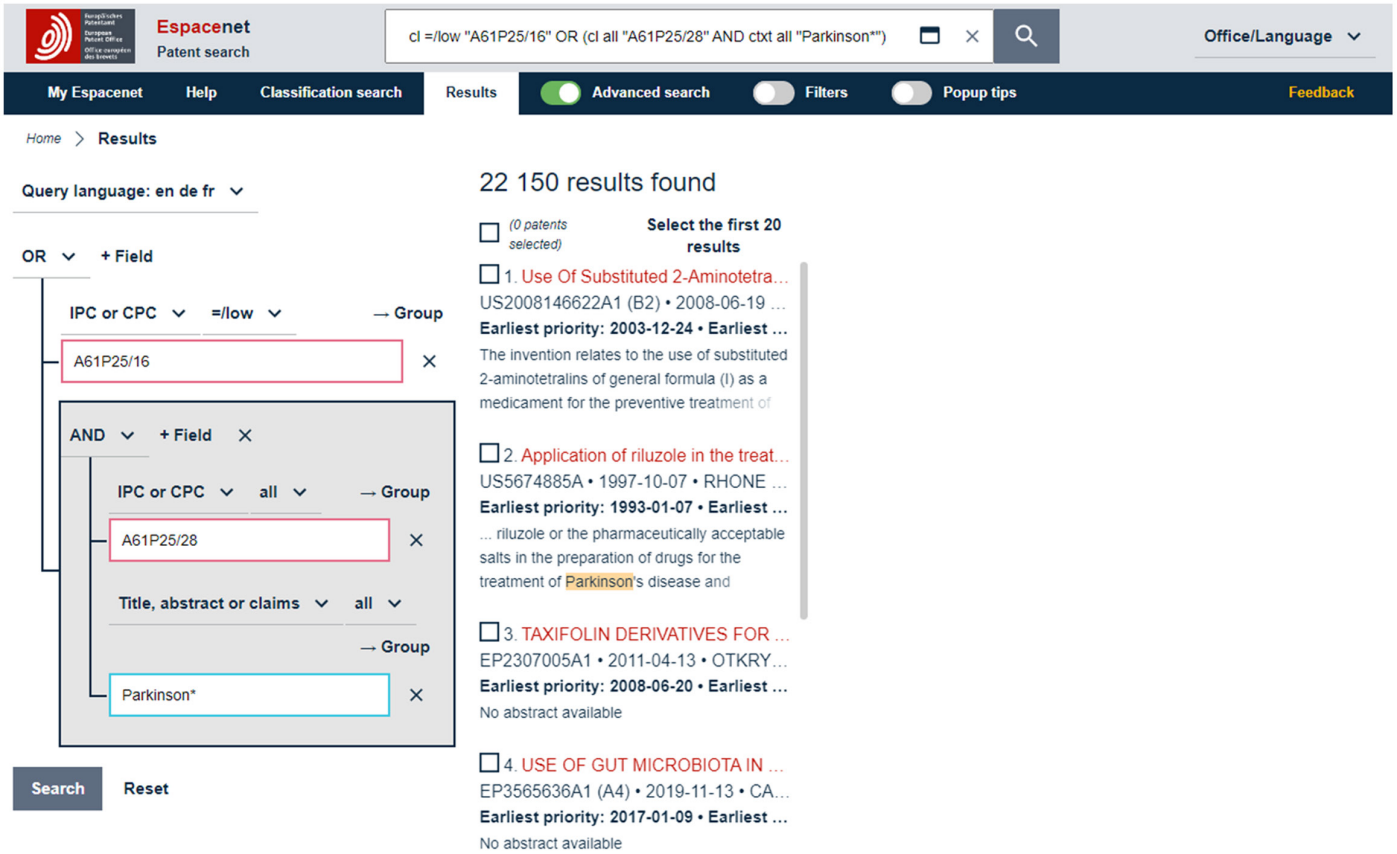
Retrieved records (*n* = 22.150) for search *A61P25/16 OR* (A61P25/28 AND ‘parkinson*’) on Espacenet (screen capture). Note: the classifications are being searched in *IPC or CPC* and the term Parkinson* in *T* *itle, abstract or claims*.

To search for patent records on Espacenet (and other databases for that matter), a solid search strategy must be created first. A search strategy is created iteratively based on a process roughly comprising of the following steps:

- Defining the technical field of interest
- Performing an explorative search to identify relevant patent classifications
- Creating an initial search strategy and analysing the quality of the results
- Improving the initial search strategy and creating a final search strategy

(i) Once the basic literature review has been concluded, and the research objective(s) of a project and main concepts (technology/technologies, target application[s] and/or population[s], etc.) to be investigated clearly defined, the technologies must be ‘translated’ into patent classification codes and, not seldom, keywords (together *search terms*).

(ii) Finding the most appropriate classifications can be achieved by running explorative keyword searches on Espacenet. For example, as shown in Figure 2, performing a *Parkinson AND drug* search using the *Title, abstract or claims* qualifier (and checking the IPC or CPC filter option → IPC subgroups) will reveal that A61P25/28 (treating neurodegenerative disorders) and A61P25/16 (abnormal movement, Parkinson) are the most frequently assigned classifications (green highlight). Figure 2 also shows that, not seldom, a range of (additional) classifications are assigned to a single patent.

(iii) However, there exists no specific classification that covers both *neurodegenerative disease* and *Parkinson*. So, in order to identify relevant patents, A61P25/28 should be combined with a keyword, such as ‘parkinson’. A preliminary complete search, therefore, might be: *A61P25/16 OR* (A61P25/28 AND ‘parkinson*’). Note: * (asterisk) represents a so‐called *wildcard* which instructs the search algorithm to include any term that begins with *Parkinson*, such as Parkinson's or Parkinsonism, see Figure 3. Also shown in Figure 3: classification codes must be searched using the *IPC or CPC* qualifier (rather than the *Title, abstract or claims* qualifier, which is, as mentioned above, used for keywords). This is because classification codes are not listed in the title, abstract or claims sections of patents.

(iv) In the iterative phase, the search strategy should be revised to include spelling variants, for example by using wildcards (as alluded to above) and synonyms, for instance, shaking palsy—shown, as an example, in Table 2. But other classifications can be relevant, such as those for treating the side effects of Parkinson's disease, that is A61P29 (anti‐inflammatory agents), etc.

Performing multiple explorative searches, analysing the search results, and iteratively developing a table that collates—for all technical aspects of the search topic—classifications and/or keywords, provides for the most adequate final search strategy. Creating more than one final query might also make sense. For instance for Parkinson's disease, Table 2 shows three queries for drugs targeting Parkinson's disease (a) as a movement disorder, (b) as a neurodegenerative disease, and (c) that have anti‐inflammatory effects (to treat symptoms). The number of queries that are required depend on the topic and research question.

**TABLE 2:** Queries and their combination covering the concepts *neurodegenerative disease and Parkinson*.^a^

(Drugs against →)	(a) Parkinson's disease	(b) Neurodegenerative disease	(c) Anti‐inflammatory
Classification	A61P25/16	A61P25/28	A61P29/00
Keywords		Parkinson* Shaking palsy	Parkinson* Shaking palsy

*Note*: Based on the table, a complete search would consist of: a‐classification OR (b‐classification AND b‐keywords) OR (c‐classification AND c‐keywords).

^a^ The search shown is an example and does not aim to be complete.

As there exist a vast number of patent classification codes and, as alluded to above, various code levels, we strongly advise to consult with the local/national patent office or (university) libraries to identify the appropriate, corresponding classification codes, and, ideally, keywords and Boolean operator combinations thereof, to create a sound search strategy—see, for example, *Espacenet Pocket Guide* (IV in useful resources table) and network of patent libraries (patlib centres) that exist in European Patent Office member states (useful resources V).

In addition, delimiters, such as the jurisdiction(s) included in, and time frame of the search, must be determined (and well justified). When determining the timeframe delimiter, it might be important to consider that patent applications are usually submitted to patent offices before associated research is submitted to scientific journals. This is because inventions described in scientific publications are considered publicly disclosed *prior art* and, therefore, disqualify as *novel*—one of the necessary criteria of patentability (see e.g. Latimer, 2004).

While developing the search strategy on Espacenet, search terms (keywords and classification codes) and combinations thereof can be tested, and the results reviewed conveniently by using the *Advanced Search* option—shown in Figures 2 and 3 as the fifth menu item in the blue bar at the top (right‐hand of the *Results* menu item). *Advanced Search* opens a variety of fields in which search terms can be inserted and combined with Boolean operators, qualifiers, delimiters, etc.

## PILOTING THE SEARCH STRATEGY

**FIGURE 4:**
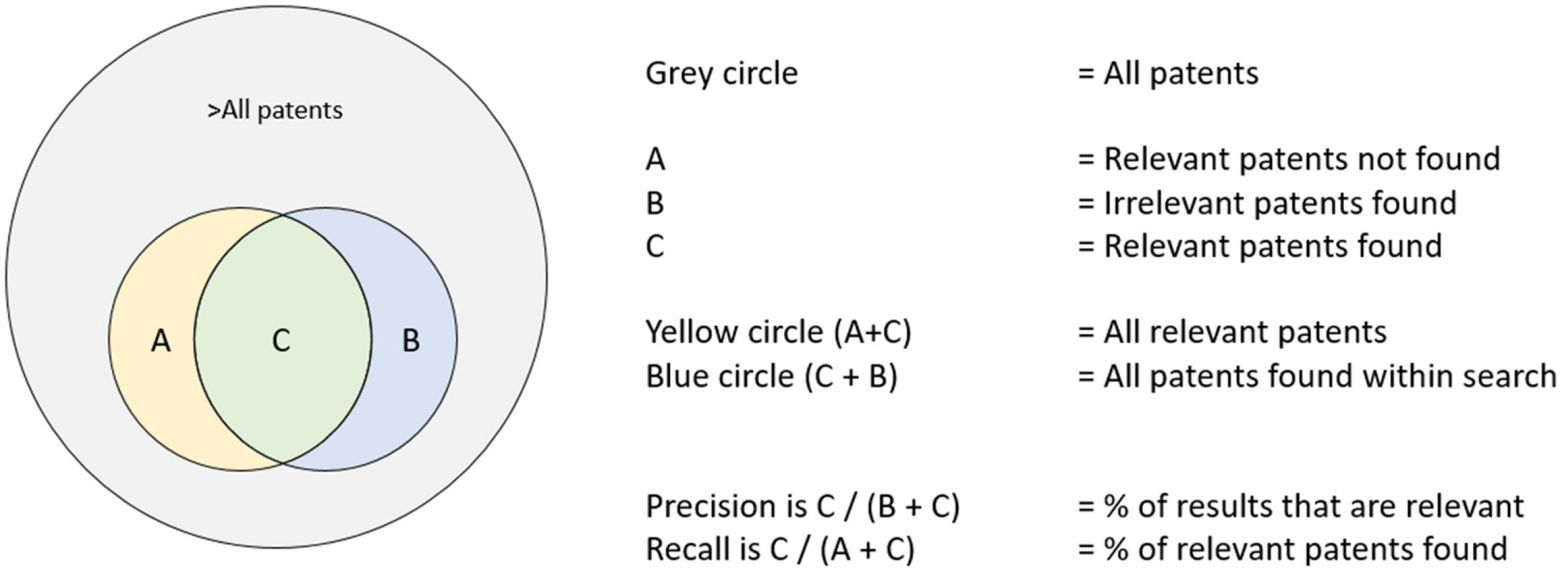
Visual explanation of precision and recall in patent searches.

Once the preliminary search strategy has been finalised (according to the procedure as explained above), it should be piloted on Espacenet, and a subset of patents reviewed to gauge the balance of the search strategy's *precision* and *recall*. A search strategy's precision refers to the ratio of relevant vis‐à‐vis irrelevant records retrieved by the search strategy; *recall* refers to the ratio of relevant records available on Espacenet vis‐à‐vis those de facto retrieved by the search strategy, see Figure 4.

Precision and recall are often somewhat difficult to balance. In basic terms: the higher the recall of a search strategy is, the ‘wider the net is cast’ and, therefore, the more relevant (but also irrelevant) records might be included; thus also potentially leading to less precision. Although proper statistical analysis is superior, quasi‐randomly selecting a sub‐sample of retrieved records – based on the number needed to screen (see below) – and carefully reading the titles and claims sections (on Espacenet) to assess the relevance of the sub‐sample for the research objectives, can provide a point estimate of the degree of the search strategy's specificity. The number needed to screen can be determined with the help of a sample size calculator, such as (Qualtrics, 2020), using, for example, a 95% confidence interval and a margin of error of 5%—if a statistician is not at hand. Recall is more difficult to assess as one might never be sure as to how many relevant patents for a specific search exist (whether on Espacenet, or other databases for that matter). However, the recall percentage can be roughly estimated based on simple calculations (Moehrle, 2018). The required percentages depend on the size of the search. Trends within small studies are easily distorted by irrelevant results, thus requiring a higher degree of *precision*. PLAs are often larger studies, so the focus might lie on increasing *recall*. A rule of thumb could be 90% recall and 70% precision ratios, as suggested in one WIPO document (Trippe, 2015). In other words, PLAs strive to be as complete as possible, but the final results of a search will never be 100% perfect. However, as is the case in all scientific investigations: validity increases when the margin of error decreases. Once precision and recall of the search strategy are within acceptable limits, the study can be continued, and individual retrieved records and filters metadata downloaded, see next section.

## USING ESPACENET FOR SMALL OR EXPLORATIVE SEARCHES

**FIGURE 5:**
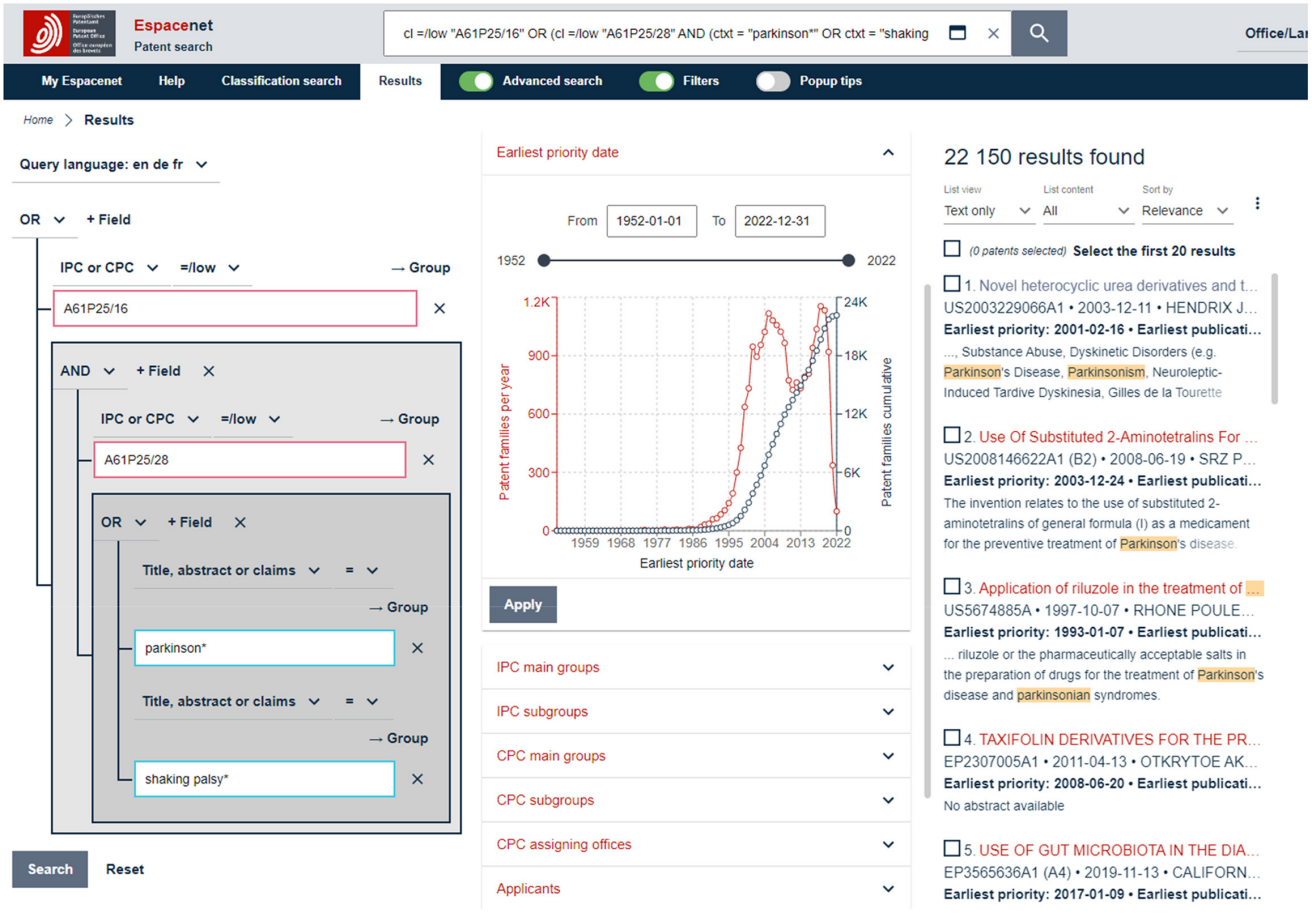
Output on Espacenet showing the result list with patent families (right) and the activated *filters panel* (centre).

As mentioned above, Espacenet was not initially designed with PLAs in mind. However, based on basic knowledge of quantitative analysis, appropriate data preparation (see examples below) and sufficient Microsoft Excel skills, it is a great first option for performing exploratory searches and basic PLAs. On Espacenet, syntaxes of finalised search strategies can be inserted directly into the search field at the top and executed, as shown in Figures 2, 3 and 5.

**FIGURE 6:**
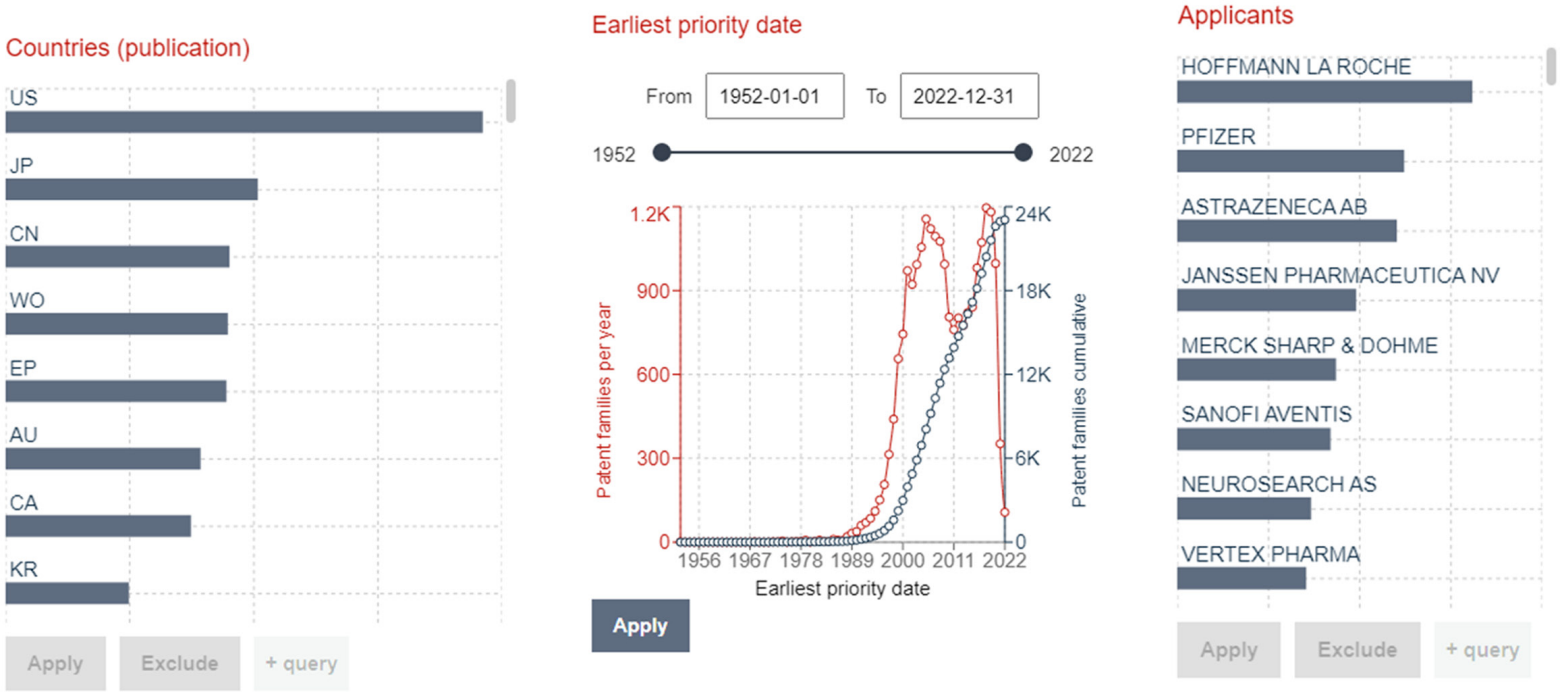
Subset of filters providing an overview of the search. Corresponding data can be downloaded as an Excel file.

There exist two ways of displaying (and downloading) patent data from Espacenet:

- As shown in Figure 3, after running a search, Espacenet initially, and per default setting, displays results as a list of patent families (in this case 22,150 results found, i.e. *retrieved records*; based on priority filing, so deduplicated)—the first display option so to speak. Here, metadata and detailed information, including claims, can be examined individually for all retrieved records. The list can be downloaded by (a) ticking the boxes next to individual patents of interest and/or (b) accessing the three vertically aligned dots that appear above the retrieved records list (right hand of list sorting options above the results list), which permits aggregate download of retrieved records. Downloaded data include metadata  only (albeit for each downloaded record), so textual analysis of claims data is not possible based on the downloaded dataset. However, this is not an issue for the method referred to here because, as mentioned above, PLA is performed on patent metadata to provide a top‐level view of technology fields and not granular analysis of the content of individual patents.^4^
- The second display option for exploring results is accessed by activating *Filters* (right‐hand of *Advanced Search* option), see Figure 5. This option provides aggregate metadata describing, and some basic statistics visualising, the composition of the body of retrieved records, for example the number of submissions per year, number of applications submitted to a specific patent office, frequency of classifications assigned within the search results (see Figure 6). *Filters* can be viewed in two modes: for priority filings (by selecting *Publication*; above the *Filters* results) or for simple families (*Family*). The *Publication* mode is deduplicated, as is the case for the default results list view; the *Family* mode displays the composition of the body for simple families as it includes all patent documents submitted per invention within the respective priority year, so not deduplicated. In many cases, the Family mode is most useful, as PLAs are often based on unique inventions that are represented by a group of documents summarised in a patent family. Anew, by accessing the three vertically aligned dots above the *Filters* results, aggregate meta‐data can be downloaded.

Regarding the first (default) results list view of retrieved records (patent families based on priority filings): one limitation of Espacenet is that, although all (e.g. more than *n* = 500) retrieved records can be displayed per search, a maximum of *n* = 500 retrieved records can be downloaded at once. Should the number of retrieved records be >500, this can be dealt with by segmenting the number of retrieved records into smaller datasets using the timeframe delimiters and running multiple searches for different time frames. An indication as to which time frame segmentation will result in fewer than *n* = 500 retrieved records per search can be elicited by viewing the temporal distribution of patenting activity in the *filters* display option *Publication Date* (*publication*) for the entire body of retrieved records (Publication mode). Time frame segmentations based on years, quarters or even months – or a combination thereof – might be necessary. Once all data for priority filings (based on default list view) have been downloaded, the datasets can be merged – in case segmentation has been necessary. There exist no limitations regarding the download of data for the *Filters* option.

**FIGURE 7:**
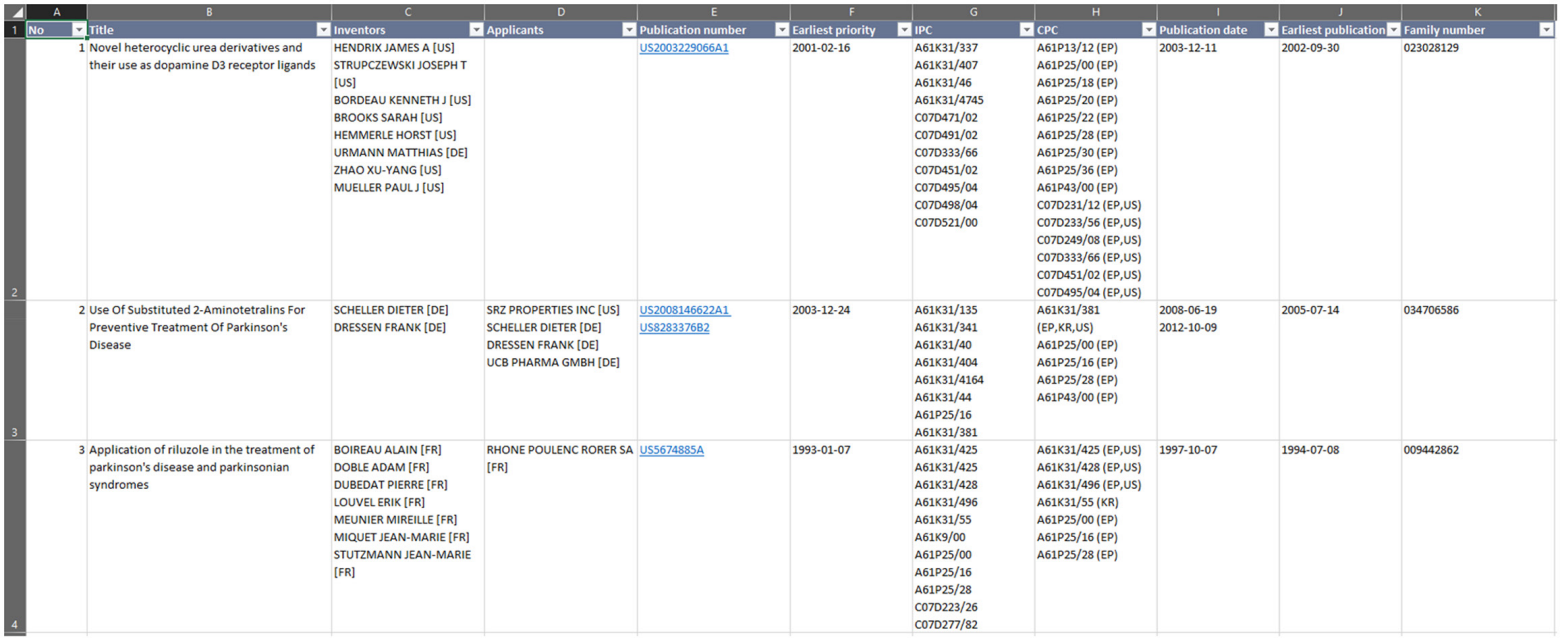
The Excel output of the results list, showing information per patent family. Data manipulation should be done before analysis.

The explanations hereafter assume that primarily automated data manipulation will be employed to prepare, augment and analyse the datasets—first and foremost on the (merged) priority filings dataset. Anew, the latter is the dataset based on the default display option (list results), see Figure 7 for an example of a resulting dataset.

## DATA PREPARATION

First of all, backups of the downloaded datasets—which have not been modified and together with the search queries upon which they are based—should always be held separate from the datasets that are the work in progress (WiP). A very important rule to start with: patents are always associated with *family numbers*. The family number should—at all times—remain the unique identifier for each patent (data line). In some cases, analysis might require duplication of data lines (preserving the identical identifier per patent) if additional information is added to the dataset to differentially explore associations/distributions across applicant groups, technologies, etc.

If working with MS Excel, as an alternative to a DBMS, it is highly recommended to re‐organise the data into a Pivot table format early on. During this process, relevant data across the tables can be collated into one master table. Using a Pivot table substantially reduces the need for manual coding of formulae for sorting and filtering data, and advanced analysis. Manual coding can be a highly time‐consuming undertaking and is easily underestimated.

Irrespective of the decision to use Pivot tables or not,^5^ in a first step, the WiP datasets must be cleaned and prepared for proper analysis: some data columns will have to be transformed and harmonised; others augmented and associated with new variables; even others omitted, if these prove to be irrelevant for the investigation; etc. Just to re‐iterate, data manipulation depends on the type of data and analysis that is required per the research objective. While minute explanation of this process is beyond the scope of this communication, some basic examples of data transformation and/or augmentation are provided below.

The *Applicants* data column provides, on one hand, the names of entities that have applied for patents (often one organisation per patent but can also be multiple organisations and/or individuals) and, on the other, the applicant's country of origin—in square brackets. To be able to, for example, determine where R&D is likely being performed, these two pieces of information must be separated and the country of origin placed in a new column, for instance *Applicant country of origin*. Similarly, the first two letters of the *Publication Number* data column indicate to which patent office the application was submitted. Moving these two letters to a new column, for example *Receiving patent office*, can support analysis of which jurisdictions (and markets) might be considered particularly attractive to applicants. A slightly more time‐consuming task can be the association of applicants with different types of applicant groups (clusters), such as academia, private sector, and public–private partnerships. In this case, keyword‐mapping (Ltd., GmbH, SPA, etc.) and carefully selected truncation including wildcards (e.g. Universi*) and an Excel formula that automatically fills‐in a new, separate column, for instance *Stakeholder*, based on decision rules—for example *Ltd*. found → *private sector* inserted on same line in the newly created column *Stakeholder*—can be very helpful. Naturally, as designations can be misleading, keyword‐mapping requires judicial testing and multiple iterations, manual searches and revision, and diligent validation to be accurate.

One of the most important data transformations regards technology classifications (IPC and CPC codes). It is critical to be aware of the scope of each relevant classification, as technology classifications cover a broad array of different concepts that often are analysed separately and, later, recombined/cross‐referenced at an aggregate level. The IPC column features classification codes only, whereas the CPC column includes classifications and their jurisdiction(s) of application (also in square brackets) that might need to be separated – as described for instance for *Applicants* above. While data on CPC‐dependent jurisdictions of application introduce further interesting granularity to the investigation, the combination and examination of these data concomitantly add a further layer of complexity to the analysis. In the vast majority of cases, multiple classifications (and classification levels) are assigned to patents. Therefore, the *IPC* and *CPC* columns feature multiple classification codes (including main groups and sub‐groups) per cell/entry which are separated by a line break in Excel. For proper analysis, the classification codes (and, if applicable, jurisdictions of application) must be separated into individual columns or rows. Basically, the line break between the classification codes must be replaced by a comma and, in a second step, the codes separated into individual columns. Transformation to separate rows requires knowledge of Excel macros. Different commands can be used with Excel to perform these sorts of transformations—some can be accessed conveniently via the Excel menu bar. Given the amount of available data and effort required to clean the data, PL analysts must be clear about how they would like to use and analyse these differential data early on. This reduces the amount of work in the later stages of the study.

## DATA LABELLING

Moreover, as is the case for applicants, meaningful clustering of classification codes according to important concepts of the study in question is an important step to sort and make sense of the data. Two basic examples for health technologies: classification sub‐classes C12Q1 (*Measuring or testing processes involving enzymes, nucleic acids or microorganisms*) and G01N (*Investigating or analysing materials by determining their chemical or physical properties*), basically, both cover technologies that are classified as being diagnostics or having diagnostic properties. Should diagnostic purpose/use in general provide sufficient detail for analysis, one might add a further column, for instance *Diagnostics* that can take the dichotomous values *Yes* or *No*. This summarises all C12Q1 and G01N codes and subcodes (notwithstanding a priori proper separation as alluded to above) and simplifies subsequent analysis based on the new column (using dichotomous values rather than a range of classification codes).

On the other hand, likely only subsets of all classifications assigned to a patent will be interesting for analysis. The sub‐class classification codes A61P (*specific therapeutic activity of chemical compounds or medicinal preparations* [i.e. basically, the stratification of technologies based on target disorders]) and A61K (*preparations for medical, dental or toilet purposes*), or sub‐codes thereof, are usually assigned together, but not all (sub‐)codes might be relevant for a study. If, for instance, *Bacteria* (*therapeutic use of a bacterial protein*) (A61K35/74) as a therapeutic entity is to be investigated for two disease targets—say *Drugs for disorders of the metabolism* (A61P3) and *Drugs for disorders of the endocrine system* (A61P5)—one might cluster the two A61P codes using the same method as described for diagnostics and assign *irrelevant* to the remaining A61P codes in the dataset. In any case, meaningful clustering depends heavily on careful (and meaningful) consideration. Finally, it is absolutely crucial that the rationale for, and steps taken to arrive at, specific data clusters are judiciously documented, convincingly justified and transparently reported (study background, main concepts and objectives as well as the scope of associated classifications).

## DATA ANALYSIS

As is the case for other types of studies, first, descriptive analysis should be performed summarising the overall body of data. The goals of the research project will then determine the course of inferential analysis. The latter will have been performed partially whilst cleaning, augmenting and clustering data, as alluded to above. Various associations can be explored, in particular with reference to the technology classifications, such as comparing the (relative) growth rates and compounded annual, i.e. geometric, growth rates (Javorsek, 2015), of patenting activity for different technology classes and in relation to types of applicants and/or their locations, receiving patent offices (their locations and patent applicability), etc., and importantly, combinations thereof, across the search timeframe. To calculate geometric growth rates, the RRI function of MS Excel can be utilised. There are a host of other analyses that can be performed, such as patent quality based on, for instance, indicators as suggested by Neevel et al. (2020), granting status of patents,^6^ and so on. For more general information on types of analyses, refer to (Bubela et al., 2013; Clarke & Jürgens, 2019; Jürgens & Clarke, 2019; Trippe, 2015). Although we have not quality appraised the following studies, they do provide some (basic) examples of how patent data can be analysed and visualised (Bencze et al., 2019; Mahlia et al., 2020; Mendes et al., 2019)—refer to the results sections.

## APPLICATION OF PLA: SOME FINDINGS FROM OUR RECENTLY PERFORMED PATENT LANDSCAPE ANALYSIS

Although the human microbiome is considered highly promising as a source for, and target of, new health interventions, many central aspects—from basic terminology and nomenclature, over laboratory sampling and diversity assessment techniques, to trial designs and safety and efficacy measures—remain a matter of extensive scholarly debate. Publication of discoveries in this field has increased significantly since 2013. Apart from faecal microbiota transplant, which has exploratory—IND (Investigational New Drug)—status for treating recurrent *Clostridium difficile* infection as a last line treatment in the hospital setting, to date no microbiota‐based live biotherapeutic products (LBPs) that claim de facto health benefits have received marketing authorisation by the EMA nor FDA. This is not further surprising: due to the pervasive uncertainty in the field, regulatory guidelines and approval procedures in (and across) the EU and US are not yet adequately formulated to deal with appraisal of microbiota‐based LBPs, nor harmonised. This can act as a major deterrent for healthcare innovators weighing the costs of developing pertinent new interventions vis‐à‐vis the probability of recouping their expenses and realising financial rewards and thus lead to innovation frustration, and, in turn, other serious adverse consequences for patients and health systems. In order for research funders to prioritise scientific enquiry into knowledge gaps pertinent to regulatory oversight, overviews showing technologies that, potentially, will require regulatory review in the near‐ to mid‐term future are crucial. PLAs can provide an important contribution in this regard.

In mid‐2020, we performed a patent landscape analysis (timeframe 2013–2018, as patent applications are published 18 months after submission) to determine the patenting activity trajectory of microbiome targeting interventions across different disease clusters. We found that, in absolute terms, annual increases in patenting activity existed across diverse disease areas (most prominently and not surprisingly for technologies targeting disorders of the alimentary/GI tract) for the entire period. However, initial substantial annual increases, in relative terms (year‐on‐year), shifted in 2016 to significant relative annual decreases indicating declining interest in protecting (and thus potentially a priori developing) novel health technologies based on, or targeting, microbiota. Nevertheless, we also found that three disease clusters showed important recent (relative) increases, namely technologies targeting cancer, and disorders of the (neuro‐) muscular and respiratory systems. These increases were led by the private sector. Although we did not provide detail on technologies filed per individual applicant, that is specific organisations, this would have been possible. Finally, we also found that, while a large share of patent applications originated in the United States, South Korea, Japan and Europe, the by far largest proportion of patents were received by China, relative to other receiving patent offices. This is not further surprising as China's patentability and reimbursement rules are more favourable. Based on review of the results of our PLA, public and private R&D organisations, innovation policy makers, arms‐length bodies (e.g. regulators) and pertinent funding entities can assess and confirm or re‐focus, individually and ideally collaboratively, their priorities and incentives for improving the availability of and accessibility of sustainable and societally relevant microbiota targeting healthcare interventions. Further details and implications of our work can be reviewed in (Timmis et al., 2022).

## OUTLOOK

Although gradually recognised (also by academic groups), patent information and the analysis thereof, for the most part, remain underappreciated within academia, and even parts of the private sector—especially among academic entrepreneurs and small and medium enterprises. However, PLAs can significantly reduce information asymmetry and, in turn, provide highly relevant decision information for academia and policy makers based on the utilisation of relatively few resources. Outcomes of PLAs can prevent replication of research that has already been performed and thereby reduce the waste of limited resources, reveal gaps in the body of knowledge and the translation thereof into novel processes, products and services, but also focus research efforts on areas that remain under‐explored. If horizon scanning, for instance for new health technologies, is the goal of a PLA—by the private sector, or heath technology assessment and other arms‐length bodies, etc.—PLAs might be repeated on a regular, for example annual, basis. Provided no external shocks or major discoveries occur and should the ultimate goal of a PLA be to inform mid‐term policy (e.g. regulation) and research funding priorities, the conclusions of a properly executed PLA will likely be valid for a significantly longer period of time. Naturally, this depends on the general dynamics of the sector under investigation. It is even conceivable that, therefore, basic PLA should become a standard component of pertinent grant applications in the future, in order to maximise the purposeful allocation of public funds. The above‐mentioned rationale is not only valid for the public sector, but also for R&D‐driven companies. Using patent information in early R&D stages focusses attention on research gaps and, thereby, potential new avenues for scientific enquiry, affords the potential to advance existing technologies (the state of the art) and develop impactful innovations and, as a consequence, provide new or better solutions to hitherto unaddressed (societal) challenges.

**Table:** 

**Glossary**	
Applicant	The individuals(s) but more often organization(s) applying for a patent.
Application	The initial submission/filing of a patent at a patent office. Each application receives a corresponding application number. All publications of a single patent office are linked to the application.
Inventor	The individual(s) who ideated/created the invention described in a patent.
Patent	An intellectual property right, granting its proprietor the right to prevent third parties from commercially using an invention without authorization.
Patent classification	In order to render patent searching feasible/easier, patents are classified using an extensive classification system based on diverse technical areas. One or more classifications are assigned by national patent offices. Amongst others, the International Patent Classification (IPC) and Cooperative Patent Classification (CPC) are widely used.
Priority filing/application	The first patent application submitted within a simple patent family (see below). The application filing date is referred to as the priority date.
Publication	Any patent publication, often application documents or patent grants. All publications have a unique publication number, starting with a country code, a number, and a kind code that describes the type of publication, for example EP#######B1 for a European (EP) granted patent (B1).
Simple patent family	A group of patent publications, containing applications, grants and/or other publications describing the same invention. All publications are linked together based on identical priority filings.

## AUTHOR CONTRIBUTIONS

**Tomas van Rijn:** Formal analysis (equal); methodology (equal); visualization (equal); writing – original draft (equal); writing – review and editing (equal). **James Kenneth Timmis:** Conceptualization (equal); formal analysis (equal); methodology (equal); writing – original draft (equal); writing – review and editing (equal).

## FUNDING INFORMATION

None to declare.

## CONFLICT OF INTEREST

None to declare.

## Figures and Tables

**FIGURE A1:**
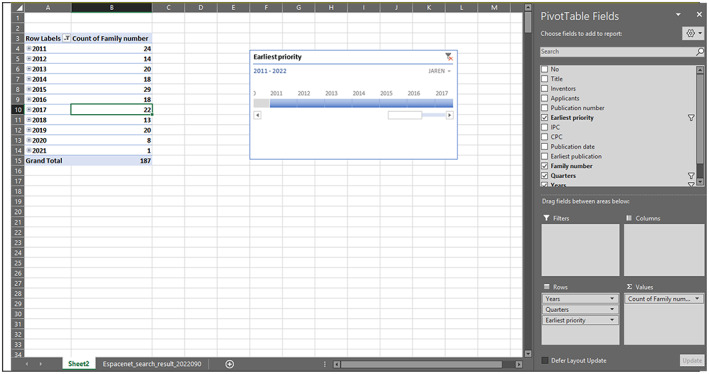
Screenshot above shows the added value of a pivot table as pivot tables allow analysts to drag and drop various variables to rows and columns. An easy example is shown for count of patent families (values) per earliest priority year (rows), but more rows or columns could be added when the dataset is well structured.

## Endnotes

^1^
This work should be viewed as a stimulation to explore patents as a source of important decision information and act as an incentive to study existing guidelines, the review of which is central to performing a PLA properly.

^2^
Note: other patent databases exist and comparisons thereof can be found in scientific publications or on the web (Jürgens & Clarke, 2018; Jürgens & Herrero‐Solana, 2015). In brief, publications often distinguish between various types of patent databases:

- national databases managed by patent authorities, like those from USPTO, CIPO or JPO, contain national patents and their legal information
- multinational databases maintained by patent authorities contain patents from multiple countries, such as Patentscope, Espacenet or Depatisnet
- commercial databases, as described above, are often multinational but access to the data and the additional functionalities is based on fee for service
- multinational databases that are not managed by patent authorities—in addition to the Lens, examples include Google patents, Patent Inspiration or Free Patents Online

For PLAs, the choice for a database is primarily determined by the data coverage for the geographical region of interest. That being said, as we provide guidance in the execution of explorative or small PLAs, familiarity and comfort with the database used may be of equal importance—for learning purposes.

^3^
While the principles of search strategy development are, in essence, independent of the specific database of choice, exploring classification codes and keywords, and creating a search strategy, as described in this communication, necessarily require extensive testing on Espacenet. Therefore, review of section *Using Espacenet for small or explorative searches* can be helpful while following the steps we describe in this section.

^4^
Nevertheless, if claims analysis of individual records is of interest at some stage in the investigation, this must be performed ‘manually’ on Espacenet.

^5^
The following explanation is based on preparing the data in the format as downloaded from Espacenet, that is before transposition to a Pivot table format.

^6^
Note: although somewhat counter‐intuitive, the granting status of a patent is not relevant for the identification of technology trends, as the application itself demonstrates the perceived commercial value of an invention for the applicant.

## PIVOT TABLES

If the necessary knowledge on how to create Pivot tables does not exist within the research team, a basic understanding can be acquired rather easily by means of perusing video tutorials that are available online. Many are easy to follow, and, within a couple of hours, the principles of re‐organising data into Pivot tables should be an accessible resource. Chat fora, such as https://www.excelforum.com/excel‐charting‐and‐pivots/, can also be very helpful. For Pivot table analysis, more in‐depth knowledge is required—this can be acquired likewise via tutorials and chat fora. However, tutorials and chat fora will not replace a team member with solid Excel pivot table experience (Figure A1).

On a practical note: to prevent double counting in pivot table analysis, the Excel function *Distinct Counts* must be used—unfortunately, this function is not available on Excel for MacOS as of yet (as far as we are aware), so a MS Windows‐based version of Excel is necessary.

## ADDITIONAL NOTES

As patent officers, for example during review of submissions for patentability and maintenance work, update/re‐classify patents, the results of any given search can change over time (marginally).
